# A High Throughput Screen Identifies Nefopam as Targeting Cell Proliferation in β-Catenin Driven Neoplastic and Reactive Fibroproliferative Disorders

**DOI:** 10.1371/journal.pone.0037940

**Published:** 2012-05-30

**Authors:** Raymond Poon, Helen Hong, Xin Wei, James Pan, Benjamin A. Alman

**Affiliations:** 1 Program in Developmental and Stem Cell Biology, Hospital for Sick Children, Toronto, Ontario, Canada; 2 Campbell Family Institute for Breast Cancer Research, University Health Network, Toronto, Ontario, Canada; 3 Donnelly Centre for Cellular and Molecular Research and Banting and Best Institute for Medical Research, University of Toronto, Toronto, Ontario, Canada; 4 Division of Orthopaedics, Department of Surgery, University of Toronto, Toronto, Ontario, Canada; Van Andel Institute, United States of America

## Abstract

Fibroproliferative disorders include neoplastic and reactive processes (e.g. desmoid tumor and hypertrophic scars). They are characterized by activation of β-catenin signaling, and effective pharmacologic approaches are lacking. Here we undertook a high throughput screen using human desmoid tumor cell cultures to identify agents that would inhibit cell viability in tumor cells but not normal fibroblasts. Agents were then tested in additional cell cultures for an effect on cell proliferation, apoptosis, and β-catenin protein level. Ultimately they were tested in *Apc1638N* mice, which develop desmoid tumors, as well as in wild type mice subjected to full thickness skin wounds. The screen identified Neofopam, as an agent that inhibited cell numbers to 42% of baseline in cell cultures from β-catenin driven fibroproliferative disorders. Nefopam decreased cell proliferation and β-catenin protein level to 50% of baseline in these same cell cultures. The half maximal effective concentration *in-vitro* was 0.5 uM and there was a plateau in the effect after 48 hours of treatment. Nefopam caused a 45% decline in tumor number, 33% decline in tumor volume, and a 40% decline in scar size when tested in mice. There was also a 50% decline in β-catenin level *in-vivo*. Nefopam targets β-catenin protein level in mesenchymal cells in-vitro and in-vivo, and may be an effective therapy for neoplastic and reactive processes driven by β-catenin mediated signaling.

## Introduction

β-catenin plays a critical role in mesenchymal cell function, regulating cell proliferation, motility, and differentiation. Mutations which stabilize its protein level can cause the mesenchymal tumor aggressive fibromatosis [1]; its level of activity regulates scar size in wound healing [2]; and it regulates mesenchymal cell differentiation during some reparative processes such as fracture repair [3]. β-catenin is a central mediator in the canonical Wnt pathway, whose activation is initiated by the binding of an appropriate Wnt ligand to the Frizzled and low-density lipoprotein receptor related protein co-receptor complex. In the absence of an appropriate Wnt ligand, β-catenin is phosphorylated at amino terminal serine and threonine sites, resulting in its being targeted for ubiquitination and proteosomal degradation by a multi-protein complex comprising glycogen synthase kinase-3β (GSK-3β), Adenomatous Polyposis Coli (APC), and Axin. In the presence of an appropriate Wnt ligand, this multiprotein complex does not target β-catenin for degradation. β-catenin translocates to the nucleus, where in concert with members of the T-cell-factor/Lymphoid-enhancer-factor (Tcf/Lef) family, regulates transcription in a cell type specific manner [4].

Cutaneous wound healing reestablishes the barrier and mechanical properties of skin, and progresses through overlapping inflammatory, proliferative, and remodeling phases. During the proliferative phase of wound repair, mesenchymal cells accumulate to reestablish the mechanical properties of the injured tissue. These cells are also responsible for the size of the scar that ultimately forms [5], [6]. Data from genetically modified mice in which β-catenin level regulates scar size, and from observations in patients in which hypertrophic scars are associated with high levels of β-catenin [3], show that β-catenin regulates the number of mesenchymal cells that accumulate during the proliferative phase of wound repair and ultimately scar size [2], [7], [8], [9], [10]. Disordered would repair, such as occurs in hypertrophic scars, lead to loss of function (e.g. limited joint motion) and have major psychosocial implications (e.g. disfigurement) [11], resulting in significant health problems [6], [12].

Aggressive fibromatosis (also called desmoid tumor) is a locally invasive soft tissue tumor comprised of mesenchymal fibroblast-like spindle cells [13]. It occurs as either a sporadic lesion or a familial syndrome, such as familial infiltrating fibromatosis, or familial adenomatous polyposis [14]. β-catenin stabilization is a universal occurrence in aggressive fibromatosis, and in most cases it is caused by a somatic mutation in β-catenin removing an amino terminal serine or threonine phosphorylation site, although in familial cases it is associated with a germline mutation in *APC* [15], [16], [17]. β-catenin stabilization is sufficient to cause aggressive fibromatosis as shown using a transgenic mouse model that over-expresses a stabilized form of β-catenin [9]. Aggressive fibromatosis is problematic to treat, with recurrence frequently occurring after chemotherapy or surgery. Because of the cytologic similarity to hypertrophic scarring and a role for β-catenin in both processes, aggressive fibromatosis is sometimes thought of as unchecked wound repair [18].

There are no universally effective pharmacologic approaches to manage these β-catenin driven fibroproliferative disorders. One strategy to identify an effective pharmacologic treatment is to examine libraries of diverse compounds for a potentially efficacious agent. Libraries of such compounds in which pharmacokinetic and toxicologic data is already available, makes the translation to patient care potentially easier [19]. Here we report on the use of this screening approach to identify a compound that targets cell viability driven by β-catenin in mesenchymal cells, and tested its ability to decrease scar size in cutaneous wound repair and as a therapeutic approach for the tumor, aggressive fibromatosis.

## Results

### A compound screen identified Nefopam as an agent that inhibits viability in cell cultures from aggressive fibromatosis and hypertrophic cutaneous wounds

**Figure 1:**
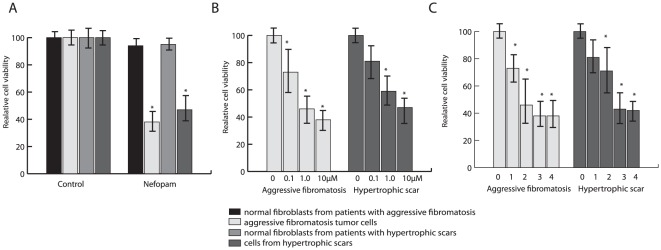
Nefopam inhibits cell viability in cultures from aggressive fibromatosis and hypertrophic cutaneous wounds. Data from the Sulforhodamine B assay. The mean value for the cell culture treated with carrier was arbitrarily defined as 100 for each cell type. A) Data for a dose of 10 µM of Nefopam. There was a significantly lower level of cell viability in the aggressive fibromatosis or the hypertrophic scar cultures compared to that seen in normal tissues. B) Data from different doses of Nefopam in cell cultures from aggressive fibromatosis or from hypertrophic scars, showing a dose dependent decrease in cell viability. C) Time course data for cell viability after days of treatment with 10 µM of Nefopam in cell cultures from aggressive fibromatosis or from hypertrophic scars. Data is given as means and 95% confidence intervals. Control data is generated by treatment with diphenhydramine. Treatment with carrier alone gave identical results as for diphenhydramine. An asterisk over a data point indicates a significant difference form the control cell cultures.

To identify potential agents that could be used to target mesenchymal cells in which β-catenin is activated, we examined primary cell cultures from aggressive fibromatosis and normal fibroblast cultures from the same patients, as well as from primary fibroblast cell cultures from hypertrophic scars and normal fibroblasts from the same patients. Cell cultures were prepared as previously reported [8], [16]. The cultures were screened using the MicroSource Spectrum collection, which consists of 2,000 compounds, some of which are already in use in humans [20]. Compounds were selected for further study based on two criteria: that they inhibited cell viability in cell cultures obtained from aggressive fibromatosis tumors; and that they showed little to no effect on normal fibroblast cell cultures from the same patient. The experiments were repeated in triplicate within 96 well plates, with each well containing 4000 cells treated with 0.1 1.0, or 10 µM of each compound or DMSO as a control and studies after three days in culture. The Sulforhodamine B assay (SRB) was used to measure cell viability, and a robotic system used to measure relative viability in each well. Compounds identified in the initial screen underwent further testing using six additional aggressive fibromatosis samples primary fibroblast cell cultures from hypertrophic scars from six patients. In all twelve cases normal fibroblasts from the same patients were also studied. The agent was tested at a variety of doses, and for various durations ranging from one to four days. This screening process identified the agent, 5-methyl-1-phenyl-1,3,4,6-tetrahydro-2,5-benzoxazocine, or Nefopam [21], which met the criteria in that it inhibited cell viability in cultures derived from hypertrophic wound samples and aggressive fibromatosis cell cultures, but not in normal fibroblasts from the same patients (Fig. 1).

### Nefopam inhibits cell proliferation in fibroblast cell cultures from aggressive fibromatosis tumors and from hypertrophic cutaneous wounds

**Figure 2:**
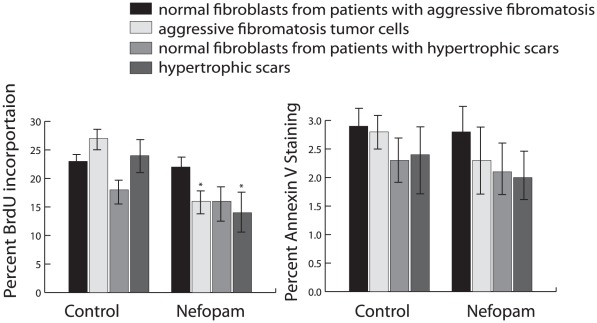
Nefopam inhibits cell proliferation in fibroblast cell cultures from hypertrophic cutaneous wounds and from aggressive fibromatosis tumors. Percent BrdU incorporation or annexin V staining from the various cell cultures treated with 10 µM of Nefopam. Data is given as means and 95% confidence intervals. An asterisk over a data point indicates a significant difference form the control cell cultures. There was a significant difference in BrdU incorporation in the tumor and hyperplastic scar cultures treated with Nefopam compared to treatment with carrier alone. Treatment with diphenhydramine resulted in the identical findings as for treatment with the carrier.

Cell cultures from hypertrophic cutaneous wounds and from aggressive fibromatosis tumors, along with normal fibroblasts from the same patients, were then examined for the effect of Nefopam on cell proliferation, and apoptosis after two days in culture. Cells from aggressive fibromatosis tumors or hypertrophic wounds treated with Nefopam had a significantly lower proliferation rate, while normal fibroblasts from the same patients did not demonstrate an appreciable change in proliferation rate, as measured using the BrdU incorporation assay [22] (Fig. 2A). There was a modest, but not significant change in apoptosis, as detected using annexin V staining [23] (Fig. 2B). Cell cultures were also treated with diphenhydramine, as a structural control since Nefopam is derived from this compound [24]. Diphenhydramine treatment had no effect on any of the cell parameters tested. The half maximal effective concentration (EC50) for Nefopam was 0.5 uM and there was a plateau in the effect after two days (48 hours) of treatment. Thus, Nefopam has predominant effect inhibiting cell proliferation, in these cell cultures.

### Nefopam reduces β-catenin protein level

**Figure 3:**
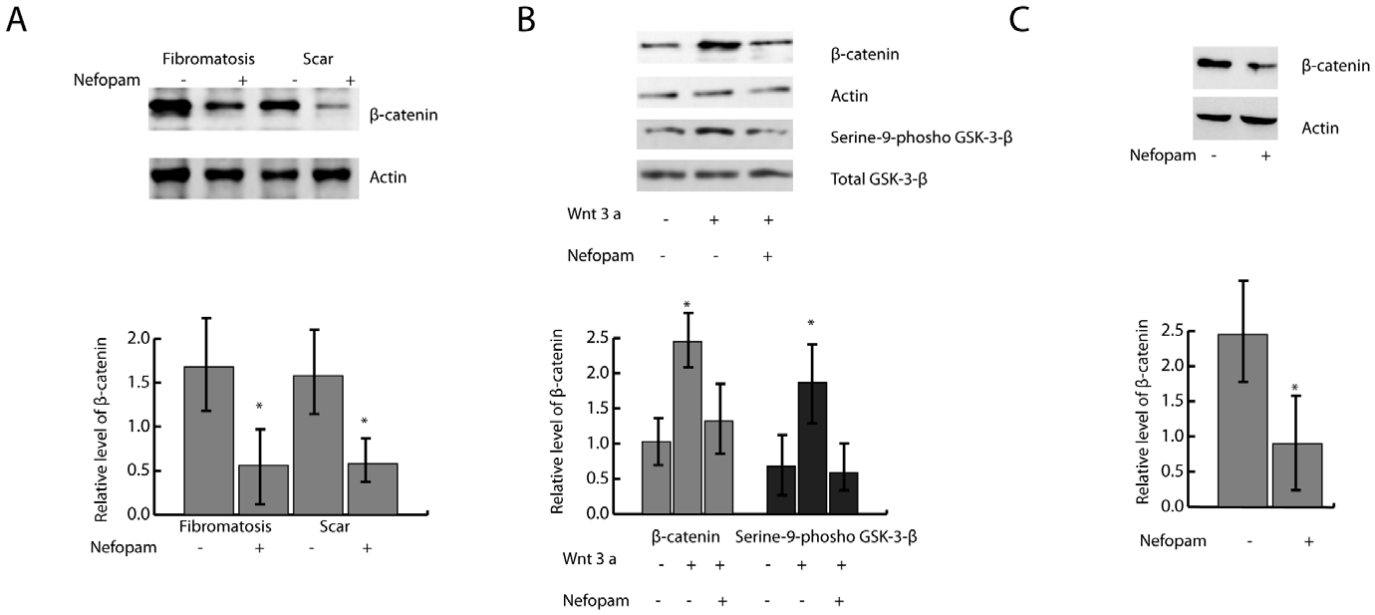
Nefopam modulates β-catenin protein level. A) Western blot analysis for β-catenin in aggressive fibromatosis and hypertrophic scar cell cultures treated with Nefopam. A representative Western blot for total β-catenin is shown at the top of the panel. The data is shown in graphical form in the lower part of the panel, as mean and 95% confidence intervals for the relative density compared to the loading control for six pairs of cultures for each condition. An asterisk over a data point indicates a significant difference form the control cell cultures. There is a decrease in β-catenin level with Nefopam treatment in cell cultures from both conditions. B) A representative Western blot for β-catenin and serine-9-phosph-GSK-3-β in normal fibroblasts treated with Wnt3a is shown at the top of the panel. Composite data is shown in graphical form in the lower part of the panel. Actin and total GSK3-β are used as loading controls. There is an increase in serine-9-phosph-GSK-3-β and β-catenin with Wnt3a treatment, which is significantly lowered, close to the baseline level, with Nefopam treatment. C) A representative Western blot for β-catenin in fibroblasts expressing a stabilized form of β-catenin lacking exon three is shown on the top of the panel. Composite data is shown in graphical form on the bottom of the panel. There is a decrease in β-catenin with Nefopam treatment.

To examine whether Nefopam has the capacity to modulate β-catenin, we studied primary cell cultures derived from aggressive fibromatosis tumors, hypertrophic wound fibroblasts, as well as normal fibroblasts from the same patients. Western blot analysis using an antibody against total β-catenin demonstrated a marked decrease in the amount of protein as a result of Nefopam treatment (Fig. 3A). To determine if Nefopam inhibits canonical Wnt signaling, we treated normal fibroblasts with Wnt3a and examined the level of β-catenin level, as well as the phosphorylation level of another canonical Wnt mediator, GSK3-β, with and without treatment with Nefopam. There was a significant increase in the level of serine-9-phosphorylation of GSK-3-β and β-catenin with Wnt3a treatment. Nefopam treatment inhibited the levels of serine-9-phospho-GSK-3-β and β-catenin back towards baseline levels when the cells were treated with Wnt3a (Fig. 3B), showing that the Nefopam targets canonical Wnt signaling cascade in fibroblasts. To determine if Nefopam affects cells expressing a mutant form of β-catenin, we examined fibroblasts expressing a form of β-catenin lacking exon three [2], which encodes the amino terminal serine or threonine phosphorylation sites. Treatment with Nefopam resulted in a decrease in β-catenin protein level (Fig. 3C). In all of the cultures examined, Nefopam did not alter the expression of β-catenin at the mRNA level.

### Nefopam suppresses the neoplastic phenotype in murine aggressive fibromatosis tumors

**Figure 4:**
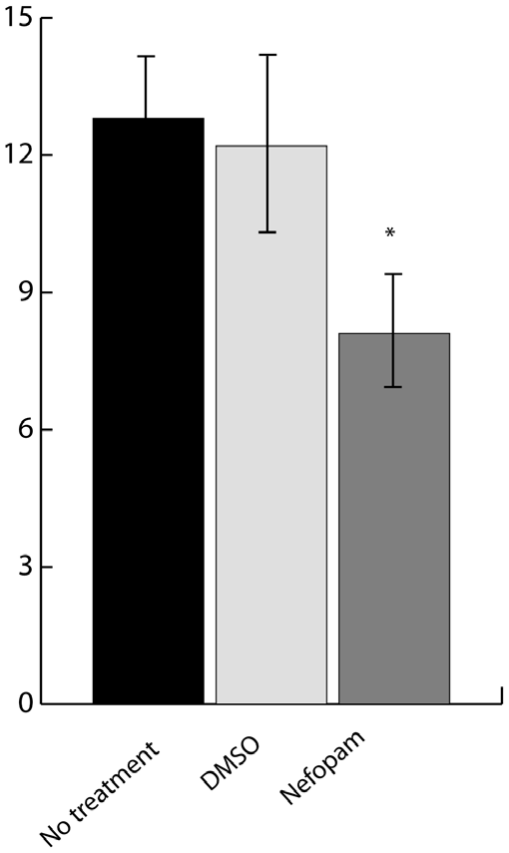
Nefopam suppresses the neoplastic phenotype in murine aggressive fibromatosis tumors. The number of tumors found in male *Apc1638N* mice. Data is given as the mean and 95% confidence intervals for the number of tumors formed in male mice in no treatment; 0.1% DMSO (carrier); or Nefopam treatment groups. Data is given as means and 95% confidence intervals. An asterisk over a data point indicates a significant difference form the control cell cultures. Mice treated with the carrier showed a comparable number of tumors to mice that received no treatment, while mice treated with Nefopam developed significantly fewer tumors.

We then investigated whether Nefopam treatment was able to modulate the phenotype of aggressive fibromatosis tumors in vivo using the *Apc1638N* mouse, which develops large numbers of aggressive fibromatosis tumors [25]. The number of tumors that developed in male mice treated with Nefopam was significantly reduced compared to the number formed in mice provided with no treatment or treated with 0.1% DMSO as vehicle or carrier control at 5 months of age (Fig. 4). There was also a 25% decrease in tumor volume.

### Nefopam regulates scar size and β-catenin levels in cutaneous wound repair

**Figure 5:**
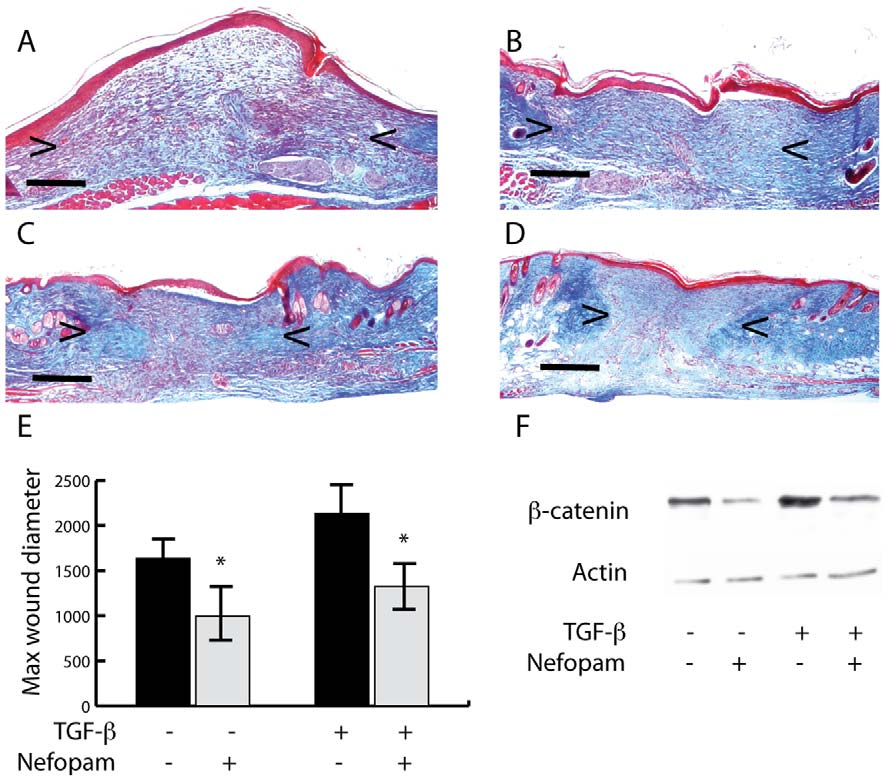
Nefopam regulates scar size and β-catenin level in cutaneous wound repair. A to D) Representative histologic sections through the widest margin of scars 14 days after wounding. A) A wound from a mouse treated with TGF-β. B) A wound in a mice treated with carrier only. C) A wound from a mouse treated with TGF-β and Nefopam. D) A wound from a mouse treated with Nefopam. Arrows show the widest width of the scar. The black line is 500 µm in length. E) A graphical representation of the mean and 95% confidence intervals of the widest diameter of the scars in µms. F) A representative Western blot mice showing the β-catenin protein levels in wounds from mice treated with TGF-β, Nefopam, or both, showing that Nefopam decreases β-catenin protein level in the wounds.

To examine if nefopam alters the phenotype in cutaneous repair, we examined mouse skin wound healing. 4 mm full thickness circular wounds were generated on the dorsal skin [2], [9]. Nefopam or control was administered daily after wounding. Scar size was determined using histologic observations of sections cut across the wound perpendicular to the skin. The section with the widest diameter of each scar was used to measure the relative scar size, as previously reported [2]. 14 days after wounding, mice treated with Nefopam had a scar diameter half that of control mice (Fig. 5 B,D, and E). To determine if Nefopam might counteract the effect of agents that cause hypertrophic scars, we examined if Nefopam would reduce the large scar size due to subcutaneous injection of TGF-β at the time of wounding. Nefopam treatment of wounds treated with TGF-β resulted in a scar size close to that of control wounds (Fig. 5A, C, E). Protein lysates extracted from the scars were assayed for β-catenin levels. β-catenin levels are higher in wounds treated with TGF-β. There was a significant decline in β-catenin levels in the wound from mice treated with Nefopam, compared to controls (Fig. 5F).

## Discussion

Here we used a high throughput screen to identity Nefopam as an agent that inhibits cell viability in mesenchymal cells in which β-catenin is activated. To test this for its effects in-vivo, we examined the mesenchymal tumor, aggressive fibromatosis, and cutaneous wound repair in mice. Nefopam suppressed the neoplastic phenotype in aggressive fibromatosis, and reduced the scar size in wound healing.

Nefopam is a centrally-acting but non-opioid analgesic drug of the benzoxazocine chemical class which was developed in the 1970s [26]. It was generated by cyclization of diphenhydramine [24]. It has an analgesic effect is stronger than aspirin, but not as strong as codeine, and has few side effects, especially as compared to opioid analgesic agents [27]. The mechanism of action of Nefopam is not completely elucidated, although inhibition of serotonin, regulation of dopamine and noradrenaline reuptake, and the regulation histamine H3 receptors and glutamate are all hypothesized to play a role in its analgesic effect [28], [29], [30]. It also acts as a voltage-gated sodium channel blocker, and this could mediate its antinociceptive effects [31].

Despite what is known about the potential mechanism by which Nefopam inhibits pain, the mechanism by which it inhibits β-catenin signaling and suppresses fibroblast cell proliferation is not clear. Since we found that the agent primarily influences cell behavior when β-catenin is activated above physiologic levels, this suggests a threshold effect. Such a threshold effect has been demonstrated for some G-protein-coupled receptors, where the intracellular proteins binding to a receptor will cause desensitization when present above a certain threshold level [32]. D(2)-class dopamine receptors exhibit such a G-protein-coupled desensitizing effect, and can also regulate glycogen synthase kinase 3 activity [33]. Since nefopam may regulate dopamine signaling, such an effect could explain the mechanism by which nefopam regulates β-catenin activity. While such a desensitizing mechanism is reported to cause a threshold effect of a drug primarily when a signaling pathway is activated, it is also possible that activation of a signaling could inhibit expression of a protein that could bind a G-coupled receptor. In this case signaling activation would decrease expression of such a protein and thus cause desensitization of a G-protein-coupled receptor. While such a mechanism is only conjecture, a similar process can play a role in other instances, such as in diabetes, where certain drugs will only have an effect in diabetic rodents [34].

Our data shows that this agent can suppress the effects of high levels of β-catenin due to activation by a Wnt ligand, a growth factor known to activate β-catenin mediated transcription, and an oncogenic mutation in β-catenin itself. Thus, it has a broad range of activity suppressing the effects of β-catenin in mesenchymal cells. It has a predominant effect in cell cultures modulating cell proliferation, and this may be mediated by the decline in β-catenin protein level, as activation of β-catenin mediated signaling stimulates fibroblast proliferation [35]. Intriguingly, Nefopam has little effect on β-catenin in normal fibroblasts, and this finding raises the possibility that the agent primarily targets cells in which β-catenin is activated above normal physiologic levels. Such a characteristic makes Nefopam an enticing therapeutic agent, as it suggests that it will have little effect on normal mesenchymal cells.

We found that nefopam primarily effects cell proliferation, having little effect on apoptosis. This results in a relative decrease in number of cells present in culture with nefopam treatment. Since the Sulforhodamine B assay measures cell viability by measuring cell number, this inhibition in proliferation results in a lower number of viable cells, despite no change in cell death. In fibroproliferative processes, such an inhibition in cell proliferation would limit the size of the associated fibrous, thus inhibiting tumor growth and decreasing scar size.

β-catenin plays a role in a wide variety of neoplastic processes [36], [37], as well as in other non-neoplastic fibroproliferative processes [18], [38], such as renal fibrosis [39], pulmonary fibrosis [40], skin fibrosis, [40] and palmar fibrosis (or Dupuytren disease) [41]. This raises the possibility that Nefopam, would have a therapeutic application in a wide variety of fibroproliferative and neoplastic conditions. Indeed, blockade of β-catenin inhibit fibrosis in some of these processes [42], [43]. Thus, Nefopam could play a therapeutic role in a broader range of conditions than the two investigated in this study.

There is a lack of pharmacologic agents which target β-catenin that can be readily used in patient care. Nefopam is an enticing agent for use for this purpose as it has already been used in patients, and found to have a strong safety record. As such, it has the capacity to be rapidly brought to patient care to aid in the treatment of difficult to manage neoplastic and reactive fibroproliferative disorders.

## Materials and Methods

### Ethics statement

This study was carried out in strict accordance with the recommendations of the Canadian Council on Animal Care. The protocol was approved by the Committee on the Ethics of Animal Experiments of the Toronto Centre for Phenogenemic and the Hospital for Sick Children. The use of human material was approved by the Institutional Review Board of the Hospital for Sick Children. Written informed consent was obtained for the use of the material.

### Human cell cultures

Aggressive fibromatosis tumors were obtained at the time of surgery. Tumor tissue and surrounding normal fascial tissue from the same patient were processed immediately after surgical excision, and primary cell cultures generated [17], [44]. Hypertrophic wound samples and dermal fibroblasts from surrounding normal skin were harvested at the time of surgery to revise the scar, and primary cell cultures were prepared as reported [8]. Monolayer cultures were cultured in DMEM supplemented with 5% fetal bovine serum and maintained at 37°C in 5% CO2. Cells were divided when confluent and experiments were performed between the first and fourth passages. The details about the patients, processing of tissues to generate primary cell cultures, and maintenance of the cells in cultures is provided in previous publications [8], [17], [44].

To identify potential agents that would target cell viability in aggressive fibromatosis, we screened the MicroSource Spectrum collection library (Discovery Systems, Inc. Gaylordsville, CT, U.S.A.) which contains 2,000 agents, for compounds which meet two criteria: 1) inhibit cell viability of fibroblasts obtained from aggressive fibromatosis; and 2) show little to no effect on normal fibroblast cultures. The experiments were repeated in triplicate within 96 well plates, with each well containing 4000 cells treated with between 0.1 1.0, or 10 µM of each compound or DMSO as a control. The Sulforhodamine B assay (SRB) [45] was used to measure cell viability. Data on Nefopam was verified using additional cell cultures. Controls included the use of a carrier or the use of a structural control, diphenhydramine from which Nefopam is derived [24]. For these experiments hypertrophic wound or aggressive fibromatosis cell cultures from additional patients were studied. Normal fibroblasts as well as the pathologic cells from each patient were tested. Cells were treated with vehicle control 0.1% DMSO with or without Nefopam (Meda Pharmaceuticals, Bishop's Stortford, UK) or diphenhydramine (Sigma-Aldrich, Oakville, Ontario, Canada) prepared each used at the same concentrations. Normal fibroblast cultures were also examined with recombinant Wnt3a [46], [47] (R and D systems, Minneapolis, MN, U.S.A.) to activate the canonical Wnt signaling pathway.

Proliferation was measured using 5-bromo-2-deoxy-uridine (BrdU) Incorporation assay. After BrdU incubation for 12 hours, cells with incorporated BrdU were identified using rabbit monoclonal anti-BrdU antibody and horse anti-mouse antibody conjugated to Alkaline Phosphatase. Presence of BrdU was detected using Alkaline Phosphatase substrate. Percentage of positively stained nuclei out of total nuclei was analyzed over 10 high-powered fields. Apoptosis was measured by annexin V staining [23] using flow cytometry to detect positively stained cells.

### Protein Analysis

Tissue samples or cells were treated with Lysis Buffer (Roche, Montreal, Quebec, Canada). Equal quantities of total protein were separated by electrophoresis in an SDS-polyacrylamide gel, transferred to a nitrocellulose membrane, and immunoblotted overnight at 4°C with primary antibodies against phospho-GSK3-β (Ser 9, rabbit polyclonal, New England Biolabs, Pickering, Ontario, Canada), β-catenin (mouse monoclonal, Upstate Biotechnology, Lake Placid, NY, U.S.A.) total GSK3β (mouse monoclonal, Transduction Laboratories, Mississauga, Ontario, Canada), or GAPDH (mouse monoclonal, Upstate Biotechnology, Lake Placid, NY, U.S.A.). Horseradish Peroxidase-tagged secondary antibodies and Enhanced ChemiLuminescence (Amersham, Piscataway, New Jersey, U.S.A.) were used to detect hybridization. Densitometery was performed using the AlphaEaseFC software (Alpha Innotech, San Leandro, California, U.S.A.). Western blotting was performed in triplicates to ensure reproducibility.

### Mouse studies

Mice heterozygous for the *Apc1638N* allele harbor a targeted mutation at codon 1638 of the *Apc* gene. Male mice develop as many as 45 aggressive fibromatosis lesions by the age of 6 months, but female mice develop fewer numbers of tumors than male mice develop. Testosterone levels influence the sex difference in tumor incidence [48]. These mice develop an average of six gastrointestinal polyps at the same age. Male *Apc1638N* mice were divided into three groups: No Treatment; carrier (0.1% DMSO); or Nefopam (40 mg/kg body weight [49]). There were ten mice in each group. Nefopan or carrier were administered daily by oral gavages, starting at 2 months of age. Treatment was continued until the mice were five months of age. At autopsy, an observer blinded to the treatment regimen scored the size and number of aggressive fibromatosis tumors and gastrointestinal polyps as previously reported [50].

For wound healing experiments, two 4 mm diameter circular full-thickness skin wounds were generated using a dermal biopsy punch (Miltex Instrument Company, York, Pennsylvania, U.S.A.) in wild type mice. Wounded mice were either treated with Nefopam, 40 mg/kg body weight [49] given daily via oral gavages, or a carrier group (10 mice in each group). At 14 days, the mice were sacrificed, and wound tissues were collected for histological examination. Scar size was measured from the histologic sections as previously reported [2], using trichrome stained sections cut across the wound perpendicular to the skin. Serial sections were cut across the scar to identify the widest diameter of each scar (mid aspect of the scar), and this section was used to measure scar size. An observer blinded to the treatment measured the scar size. An additional 16 mice were also treated with TGF-ß at the time of wounding as previously reported [2], [10], and then with either Nefopam or carrier. The wounds were examined 14 days following injury in an identical manner as for the studies using nefopam alone. The scar size was averaged between the two wounds in each mouse, and taken as a single value for each mouse for statistical analysis. Primary cell cultures were established from The *Catnb* ^tm1Tak^ mouse, which possesses loxP sites flanking exon 3 of the gene encoding for β-catenin. When exposed to Cre-recombinase, this results in the conditional stabilization of β-catenin. An adenovirus that expresses Cre-recombinase was used to induce recombination, which was verified using PCR and Western analysis. The details of the generation of the primary cell cultures were previously reported [2].

For mice treated with Nefopam, high-performance liquid chromatography was performed on serum from the mice at the time of sacrifice, using techniques as previously reported [51], to verify systemic uptake of Nefopam.

### Statistical analysis

Data in given as means ±95% confidence intervals. Means and standard deviations were compared using the t-test. The studies were performed in at least triplicate.

### Contributions of authors

Raymond Poon undertook the animal and cell culture studies on wound repair, Helen Hong the animal animal and cell culture studies on desmoid tumors, Raymond Poon, Xin Wei, and James Pan undertook the drug screens and initial characterization of the effect of on cell behavior. Benjamin Alman conceived and designed the experiments. Helen Hong, Raymond Poon, Xin Wei, James Pan, and Benjamin Alman analyzed the data. All of the authors participated in drafting the manuscript.
